# Condition Assessment of Industrial Gas Turbine Compressor Using a Drift Soft Sensor Based in Autoencoder

**DOI:** 10.3390/s21082708

**Published:** 2021-04-12

**Authors:** Martí de Castro-Cros, Stefano Rosso, Edgar Bahilo, Manel Velasco, Cecilio Angulo

**Affiliations:** 1 Intelligent Data Science and Artificial Intelligence Research Centre (IDEAI), Automatic Control Department, Campus Nord, Universitat Politècnica de Catalunya, 08034 Barcelona, Spain; manel.velasco@upc.edu (M.V.); cecilio.angulo@upc.edu (C.A.); 2 Digihub Barcelona, Siemens Energy S.L., 08940 Barcelona, Spain; stefano.rosso@siemens.com; 3 Siemens Energy S.L., Slottsvägen 2-6, 612 31 Finspang, Sweeden; edgar.bahilo_rodriguez@siemens.com

**Keywords:** artificial intelligence, autoencoder, soft sensor, condition assessment, gas turbine

## Abstract

Maintenance is the process of preserving the good condition of a system to ensure its reliability and availability to perform specific operations. The way maintenance is nowadays performed in industry is changing thanks to the increasing availability of data and condition assessment methods. Soft sensors have been widely used over last years to monitor industrial processes and to predict process variables that are difficult to measured. The main objective of this study is to monitor and evaluate the condition of the compressor in a particular industrial gas turbine by developing a soft sensor following an autoencoder architecture. The data used to monitor and analyze its condition were captured by several sensors located along the compressor for around five years. The condition assessment of an industrial gas turbine compressor reveals significant changes over time, as well as a drift in its performance. These results lead to a qualitative indicator of the compressor behavior in long-term performance.

## 1. Introduction

High reliability, all-time system availability, low environmental impact, and human safety are key points for the profitability and competitiveness in industry [1]. Operational changes related to challenging weather conditions, operational modes, set point modifications, and, especially, non-reported hash conditions are real concerns to engineers [2]. The current trend of effective management strategies from unexpected events has been sharpened to avoid major economical, environmental, and security defects [3]. Therefore, a proper health management system as well as a good condition assessment method are mandatory. Thanks to remote sensing, big data analytics, and machine learning techniques, useful tools have been developed to tackle all these issues [4].

### 1.1. Maintenance

Maintenance is the process of preserving the good condition of a system to ensure its reliability and availability for a specific period or restoring the system to its normal operating conditions [5]. Maintenance strategies can be classified into three main categories, in increasing order of complexity: corrective, preventive, and predictive maintenance [6].

**Corrective Maintenance**

Corrective maintenance is the simplest approach to deal with system conditions. It is performed only when a component of the system breaks down [7]. It is also known as unplanned, run-to-failure, or reactive maintenance [8]. As well as being the simplest approach, it is also the less effective one, as the cost of interventions and the associated downtime are usually more substantial than those with planned corrective actions taken in advance.

**Preventive Maintenance**

Preventive maintenance is regularly performed according to a plan scheduled in advance, regardless of the health status of the equipment. The plan is based on suggestions of experienced equipment manufacturers, historic breakdowns or failure data, operating experience, and the judgment of maintenance staff and technicians. Therefore, the goal of this strategy, also called planned maintenance, is to increase the availability of the system by slowing down the deterioration processes leading to faults [8]. Maintenance actions are planned on a time or usage trigger [9]: maintenance based on a time trigger, also known as time-based maintenance (TBM), includes actions that are carried out periodically, whereas maintenance based on a usage-based trigger, named usage-based maintenance (UBM), includes actions that are planned according to a process iteration such as a certain amount of production cycles. Although preventive maintenance can reduce the probability of system failures and the frequency of unplanned emergency repairs, it cannot completely eliminate the occurrences of random failures [10].

**Predictive Maintenance**

This maintenance strategy aims at detecting possible defects, based on estimate health of the equipment, and fixing them before they result in failure [11]. It attempts to avoid unnecessary maintenance tasks by taking actions reactively. Indeed, it is a proactive process which requires the development of a system model that can trigger an alarm for corresponding maintenance [12]. Several disciplines have been developed on this strategy along time, such as condition-based maintenance (CBM) [13], structural health monitoring (SHM) [14], and prognostics and health management (PHM) [15]. The most common approaches that are used nowadays in industry are based on artificial neural networks and machine learning techniques [16].

### 1.2. Industrial Gas Turbines

Industrial gas turbines for power generation are a type of internal combustion engine that converts chemical energy of fuel into electrical power. They are mainly composed of three components: a compressor, combustor, and power turbine [5]. The main function of the compressor is to provide a sufficient quantity of air into the system to ensure its proper operation. In short, the inlet air is compressed at high pressure and discharged in the combustion chamber at the required quantity and pressure. Next, the combustion chamber, which contains several combustors, is where the combustion takes place. Pressurized air is mixed with fuel and, by using burners, the internal temperature is greatly increased. The combustor must be well designed in order to provide a complete combustion process and avoid malfunctioning events. Finally, hot gas is sent to the power turbine for power generation. The turbine is responsible for converting the energy from the fluid into useful work by expanding the hot gas. The turbine is linked to the compressor via a rigid coupling. Industrial gas turbines under this study are single-shaft systems. Indeed, the turbine ensures the rotational drive of the compressor to convey a process gas.

Several causes have been found that greatly damage industrial gas turbines, giving rise to malfunctioning events and deterioration [17]. Among them, *fouling*, defined as the adherence of particles to airfoils and annulus surfaces, leads to changes in the airfoil’s shape [18]. In fact, it is the only one that can be recovered by maintenance. Moreover, several other fatigue factors could be considered as being harmful to the system performance such as the number of starts and stops, the output power set point, and so on.

### 1.3. Machine Learning Diagnosis

The way decisions are nowadays made in the industry is changing thanks to increasing data availability [19] and the development of intelligent tools for condition and fault assessment [20]. In commercial industrial gas turbines, data are captured by several sensors located along the machine and are then sent back to the engineering office where they are analyzed and processed to diagnose any unexpected event. The main purpose of this data analytics process is to improve the performance of the system to make it more reliable, all-time available, and safer [21].

Machine learning techniques employed in data analytics are nowadays able to treat large amounts of data and return online information about the machine status. These procedures are mostly used to extract information from multidimensional time series to identify hidden patterns [22]. In this study, a particular machine learning architecture is used to identify these conditioning differences called autoencoder. An autoencoder is defined by an architecture, usually in the form of artificial neural networks, that is trained to copy its inputs to its outputs [23]. Its structure is based on two neural networks: an encoder and a decoder network. The encoder network *G*, also named encoder, is defined as an encoding function z=G(x), where x is the model input and z is a set of latent variables. The decoder network *F*, also named decoder, is defined to reconstruct the encoded signal, $x^{\prime }=F\left(z\right)$, where $x^{\prime }$ are the reconstructed input signals. The parameter sets of both networks are simultaneously learned by minimizing the loss $\epsilon =d(x,x^{\prime })$ according to some distance metric [24].

The autoencoder architecture and its variants have been effectively applied to assess the condition of many different components. Xu et al. [25] used a moving window-based autoencoder (MASAE) to construct a health indicator for predicting the Remaining Useful Life (RUL) of roller bearings. A novel method based on a stacked autoencoder (SAE) and support vector regression (SVR) was introduced by Sun et al. [26] to predict the rotor deformation of an air preheater in a thermal power plant. Using a similar autoencoder structure, Han et al. [27] proposed an intelligent monitoring model based on a stacked sparse autoencoder (SSAE) to identify the combustion stability as well as to optimize the operating conditions. Furthermore, on the same component, Yan, W. and Yu, L. [28] proposed a stacked denoising autoencoder (SDAE) to accurately detect anomalies in a gas turbine combustor. Most autoencoder architecture applications are used for classification purposes to detect component anomalies and to perform fault diagnosis.

The main goal of this study is to develop a soft sensor based on this data-driven machine learning technique following the autoencoder architecture for monitoring drift in the condition of an industrial gas turbine, particularly in the compressor, using real plant data. Drift is defined as a continuous slow movement from one working condition to another, mainly due to machine life deterioration, unexpected events, workload or maintenance interventions, like washing, to alleviate the fouling deterioration [29]. Data are collected from sensors at different locations in one industrial gas turbine’s compressor. The results are obtained by modeling the industrial gas turbine’s compressor for a short-term period with a uniform regular condition in the form of autoencoder. Thereforee, the autoencoder modeling the compressor in the short-term, $AE_{M}$, is trained on regular fresh conditions M so that $\epsilon_{M}=d\left(x_{M},x_{M}^{\prime }\right)$ is minimized. Then, this model encoded in $AE_{M}$ is used into the long-term performance of the industrial gas turbine’s compressor, P, $x_{P}^{\prime }=AE_{M}\left(x_{P}\right)$. The deviation between the original data and the one obtained from the autoencoder, $\epsilon_{P}=d\left(x_{P},x_{P}^{\prime }\right)$, is related to the drift of the component [30]. It is computed using two distance metrics d(·,·): Absolute Error and Fréchet Distance. Moreover, two calculations are considered for data time-averaging: Moving Average and Incremental Window Average.

The rest of the paper is organized as follows. In Section 2, the methodology to be applied in this research is introduced, that is, a description of the selected autoencoder models, applied data processing techniques, and an exploratory analysis of the training set. Results obtained using this methodology are shown in Section 3. Several error measures and time windows are considered. Next, in Section 4, these results are discussed and conclusions are drawn in Section 5.

## 2. Materials and Methods

Machine learning is a general computational algorithmic approach currently applied in a wide range of fields for automated analytical model building. In our case, the autoencoder architecture is considered to capture the drift behavior of the compressor in a gas turbine [31].

### 2.1. Soft Sensor Model Description

**Figure 1:**
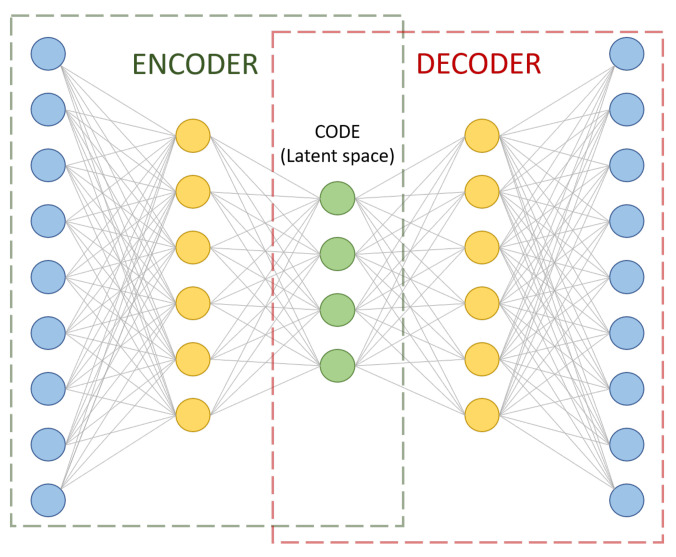
Example of an autoencoder sized according to our problem dimension.

A soft sensor can be defined as a model of a system that is based on a set of measurements that can estimate the quality variables of a process [32]. In this particular study, the model used to build the soft sensor is an autoencoder. It is an unsupervised learning algorithm that aims to set the target values so that they are equal to the original input data [33]. It is used for learning a representation or effective encoding of original data, in the form of input vectors. It is composed mainly of three parts: the encoder, decoder, and code, as shown in Figure 1. As their names state, the encoder is responsible for learning how to compress data in an encoded representation and the decoder learns how to reconstruct data that has been encoded. The code is a vector in a latent space formed from the compression/transformation of data where some hidden information can also be found.

Autoencoders can present several structures depending on the problem at hand. In this case, the most common autoencoder architecture is used. Indeed, all the nodes are connected between layers without any penalty factor in its loss. It is named fully connected (FC) autoencoder.

Regardless of the structure, there are four hyperparameters to be set before training: the number of hidden layers for the coder and the decoder, the number of nodes per layer, the code size in the latent space, and the loss function employed to measure the match between input data and recovered data. In this article, the number of layers is set to one for both the encoder and decoder. The loss function under consideration is the mean squared error (MSE) function. The number of nodes per layer is set to 6 and the code size is set to 4. Therefore, the autoencoder structure is 9-6-4-6-9, as shown in Figure 1. Finally, the activation function for all nodes is the Rectified Linear Unit (ReLU).

### 2.2. Data Processing

Data feeding the autoencoder is captured by sensors around the gas turbine’s compressor. They are placed strategically to capture their main parameters—pressure and temperature, as stated by the general gas equation (also called ideal gas law):

(1) PV=nRT

where *P* is the pressure, *V* is the volume, and *T* is the temperature. This expression can also be rewritten as

$$\frac{PV}{T}=ct$$

from where it is inferred that

$$\frac{P_{c,i}V_{c,i}}{T_{c,i}}=\frac{P_{c,o}V_{c,o}}{T_{c,o}}$$

Based on the ideal gas law, the data set has been constructed to capture derivative and proportional patterns of the main measures of the compressor. Model inputs x∈X are sampled sensors of the industrial gas turbine’s compressor. In this work, eleven features are considered, $x=(x^{\left(1\right)},\ldots ,x^{\left(11\right)})$:

- Inlet pressure (IP), $P_{c,i}$
- Inlet temperature (IT), $T_{c,i}$
- Relative ambient humidity (AH), $H_{a}$
- Pressure ratio (PR), $rP_{c}=P_{c,o}/P_{c,i}$
- Temperature ratio (TR), $rT_{c}=T_{c,o}/T_{c,i}$
- Inverse of the inlet temperature(IIT), $T_{c,i}^{-1}$
- Inverse of the outlet temperature (IOT), $T_{c,o}^{-1}$
- Inlet pressure differential (DIP), $\Delta P_{c,i}$
- Input temperature differential (DIT), $\Delta T_{c,i}$
- Output pressure differential (DOP), $\Delta P_{c,o}$
- Output temperature differential (DOT), $\Delta T_{c,o}$

The ratio features are defined as the proportion between the outlet and inlet; inverse features are included based on the ideal gas law relationship; differential features are obtained from the subtraction of two consecutive points in the corresponding time-series.

**Figure 2:**
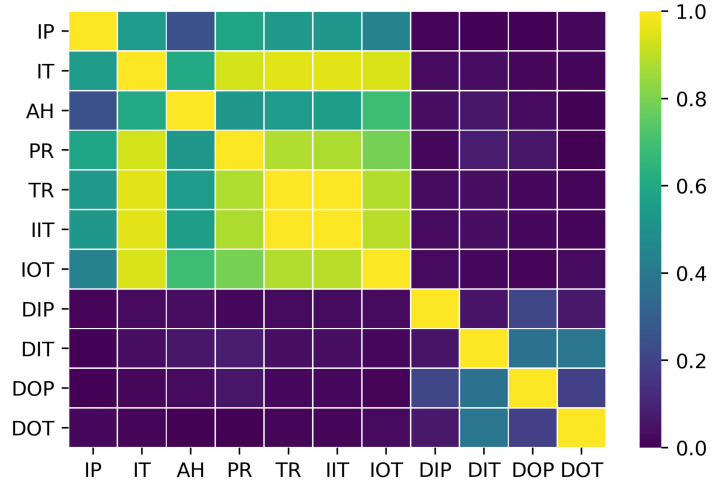
Graphical matrix representation for the values obtained from the correlation test between the 11 features considered for the data set. Each label corresponds to a single feature in an abbreviate form.

Note that they have been considered individually into the subset, that is, assuming that there is no correlation among them. A correlation test is computed in order to check whether the information given from each feature is independent. Two features are considered dependents whether they exceed a threshold in the correlation test, thus one of them is obviated. The threshold of the correlation value is usually fixed to 0.95. Nine features remain after passing the correlation test, where the temperature ratio $rT_{c}$ and the inverse inlet temperature $T_{c,i}^{-1}$ are discarded because their correlation factor is up to 0.956 and 0.957, respectively, with the inlet temperature time series. Therefore, the model input is reduced to nine features, $x=(x^{\left(1\right)},\ldots ,x^{\left(9\right)})$. Figure 2 shows graphically the results of the test.

After feature selection processing, the values of the features are preprocessed before the model training procedure. The ranges for the values in the features are large and diverse from each other. For instance, outlet pressure values can be 2000 times higher than the differential inlet pressure values. This relative variation could lead to model error because the highest values may get more influence than the smallest ones. Thus, standard scaling is used to normalize the range of independent variables or features of data with mean zero and standard deviation one.

Moreover, a filtering method is also applied for data cleaning to erase the most evident outlier elements. It is carefully defined to avoid finding out anomalies inside data. The filtering method consists of computing the median for each feature and adding or subtracting *k*-times the Median Absolute Deviation (MAD) to define both upper and lower boundaries. The median is the value separating the half of a probability distribution, while the MAD is a measure of the variability of a univariate sample of quantitative data. They can be related with the mean and the standard deviation (STD) measures; however, MAD is considered a more robust estimator in presence of outliers than STD as well as the median in front of the mean. The *k*-times constant is heuristically determined by comparing gas turbine’s sensor graphs. It has been set to seven. Therefore, the upper boundary is set to 7 times greater than the median, whereas the lower boundary is set to 7 times lower than the median.

Next, data are also filtered by the *full load* working regime. Full load is an operation mode such that the gas turbine is working at its limit condition, where degradation becomes apparent. Therefore, data are filtered at full load mode under the assumption that machine degradation can be clearly captured.

Finally, there is an operation mode called grid code regulation mode where the machine regulates the inlet air flow by itself in order to avoid harmful events when in full load. Therefore, these events are also filtered by applying a threshold in the variable guide vane (VGV) aperture. The threshold has been defined using the median of the VGV aperture in full load conditions for each equipment. Results will be presented for a single gas turbine model, the one having the highest quantity of data under the previous constraints, with training information coming from a set of ten gas turbines in the same series. In this way, we can affirm that the methodology introduced in this paper is robust enough to capture the behavior of this single model.

### 2.3. Data Set

As mentioned before, data are collected from sensors at different locations in one industrial gas turbine’s compressor for its full load working mode.

**Training Set**

The training set is the first year of gas turbine data,

(2) $$M={\left\{x\left(t_{k}^{m}\right)\right\}}_{k=1}^{N_{m}}={\left\{x_{k}\right\}}_{k=n_{m}}^{n_{m}+N_{m}}={\left\{m_{k}\right\}}_{k=1}^{N_{m}}\subset D\subset X,$$

with inputs x∈X as training features. The aim when selecting this time period for training the model is to capture the behavior of the fresh machine at the beginning of its life. This model will serve as a behavioral baseline to be compared with the performance in the long-term. In case discrepancies are found, it allows one to assess that a drift exists between the model behavior and the real gas turbine plant. Therefore, the model is defined as

(3) $$AE_{M}=\left(G_{M},H_{M}\right),$$

where M⊂X is the fresh training data.

Sampling time $T_{s}$ to generate the training dataset is 1 min. Therefore, note that $x_{k}=x\left(t_{k}\right)$ and $x_{k+1}=x\left(t_{k+1}\right)=x\left(t_{k}+T_{s}\right)$.

**Figure 3:**
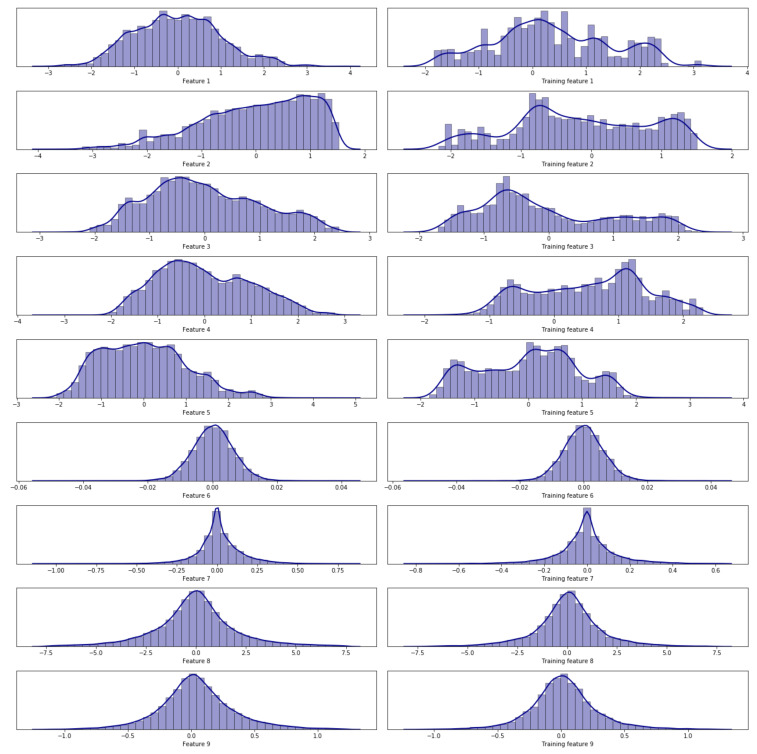
Histograms comparison between the training (**right column**) and the whole system data (**left column**).

To assess fairness in the training data, a histogram of both the training data set and the whole data set is computed. According to the number of instances, the bounds of the histogram (its maxima and minima values), and its shape, it is determined whether the training set is significant enough. In Figure 3, an example is shown. On the left column, the whole system histograms are represented, whereas on the right column, only histograms from the training data are displayed. Both are thoroughly analyzed and compared before training any model. This analysis determines whether data used in the training and the number of instances are significant enough. If so, the model would be treated as a representative simulation plant for short-term performance.

**Validation Set**

In this study, the validation set is the same as the training set. The model is constructed under the assumption that the equipment is working under fresh conditions for the first working year. It means that the working mode during this period of time is assumed as the correct one. Therefore, to validate the model, the density plot of each feature is used to ensure an entire instance representation in the training set (further explained in above) as well as the loss metric,

(4) $$\epsilon_{M}\left(x\right)=d\left(x_{M},AE_{M}\left(x\right)\right)$$

It refers to the reconstruction distance when the autoencoder $AE_{M}=\left(G_{M},H_{M}\right)$ has been trained with modeling data $M={\left\{m_{k}\right\}}_{k=1}^{N_{m}}$. It is expected that $\epsilon_{M}\left(m_{k}\right)\approx 0$, for $m_{k}\in M$, as data are reconstructed in similar conditions that the model was trained.

**Testing Set**

Test data, defined as

(5) $$T={\left\{x\left(t_{k}^{t}\right)\right\}}_{k=1}^{N_{t}}={\left\{x_{k}\right\}}_{k=n_{t}}^{n_{t}+N_{t}}={\left\{t_{k}\right\}}_{k=1}^{N_{t}}\subset D\subset X,$$

with $n_{m}\geq 1$ and $n_{m}+N_{m}\leq N$, represent the whole data set, i.e., the entire available data for the assessed gas turbine is used to test the model of the compressor to its long-term performance.

## 3. Results

The main result of the present study is a qualitative soft sensor named *Fresh Reconstruction discrepancy ($\epsilon_{F}$)* based on the deviation between the model output and the real plant values. It is defined as

(6) $$\epsilon_{F}\left(x\right)=d\left(x_{F},AE_{M}\left(x\right)\right)$$

It refers to the reconstruction distance when the autoencoder $AE_{M}=\left(G_{M},H_{M}\right)$ has been trained with modeling data $M={\left\{m_{k}\right\}}_{k=1}^{N_{m}}$. It is expected that a drift in $\epsilon_{F}\left(m_{k}\right)$, for $m_{k}\in T$, as data are reconstructed in long-term performance conditions.

To prove the drift in *Fresh Reconstruction discrepancy*, two different distances (Absolute Error (AE) and Fréchet Distance (FD)) combined with two different time-window calculations (Moving Average (MA) and Incremental Window Average (IWA)) are used, as illustrated in Table 1. Finally, deviations and trends observed using both distances and calculations are analyzed and discussed.

### 3.1. Model Distance

Model distance measures the deviation between the model and the real machine performance. As mentioned above, two distances are considered.

*Absolute Error* (*AE*) is the absolute difference between the real plant values *x* and the inferred value of the model (autoencoder) $x^{\prime }$ at each time step. Therefore,

(7) $$\epsilon_{AE}=\left|x_{i}-x_{i}^{\prime }\right|$$

The *Fréchet Distance* (*FD*) is defined as a measurement of similarity between two curves. Moreover, it can be used to measure the similarity of two distributions. In this case, the comparison is performed between two multivariate distributions—the real plant distribution and the inferred one—and is composed by nine features each and a specific time window. Assuming them as two multivariate Gaussian distributions, this distance is calculated as

(8) $$\epsilon_{FD}={\left|\mu_{X}-\mu_{Y}\right|}^{2}+tr\left(\Sigma_{X}+\Sigma_{Y}-2{\left(\Sigma_{X}\Sigma_{Y}\right)}^{1/2}\right)$$

where $\mu_{X}$ and $\mu_{Y}$ are the means of both distributions, and $\Sigma_{X}$ and $\Sigma_{Y}$ are their covariance matrices, respectively.

### 3.2. Time-Window Samples Processing

From the previously defined distance measurements, the Moving Average (MA) and the Incremental Window Average (IWA) calculations are computed to analyze the distances’ time series. The aim is to use this calculation to smooth the short-term fluctuations, including noise, and to highlight long-term trends, which capture the condition of the equipment.

*Moving average* (*MA*) is a calculation of the average of consecutive subsets of data obtained by sliding a fixed time window along the whole data set. It is defined as

(9) $$MA=\frac{1}{n}\sum_{i=0}^{n-1}x_{m-i}$$

where *m* is the current time-step, and *n* is the moving time window size.

*Incremental Window Average* (*IWA*) is a calculation of the average of cumulative subsets of data obtained by adding a fixed time window of the whole data set at each step. It is defined as

(10) $$IWA_{n}=\frac{1}{m}\left(\sum_{i=0}^{k}x_{k}+\left(m-t\right)IWA_{n-1}\right)$$

where *n* is the current time-step of the grouping subset, *m* is the length of the cumulative subsets, *x* are the instances of the current subset, and *t* is the length of the current subset.

The combinations of the two considered distances and the two smoothing time window calculations are computed for the deviation between the autoencoder and the real plant values, leading to the *Fresh Reconstruction discrepancy* and therefore the proposed soft sensor. The results are grouped according to the employed distance to make the results more understandable and easier to be compared. Therefore, the Absolute Error on Moving Average sampling (AEMA) and AE on Incremental Window Average sampling (AEIWA) are first presented, and afterwards, the FDMA and FDIWA, using the Fréchet distance, are introduced (see Table 1).

MA is calculated using multiple time windows: daily, weekly, monthly, and yearly. Assorted events and trends are shown thanks to the multiple representations of the same error. A smoother shape is observed at the largest time window (yearly window), whereas significant changes in data are observed at shorter time frames (daily and weekly windows). These allow us to tackle the analysis from several perspectives.

**Figure 4:**
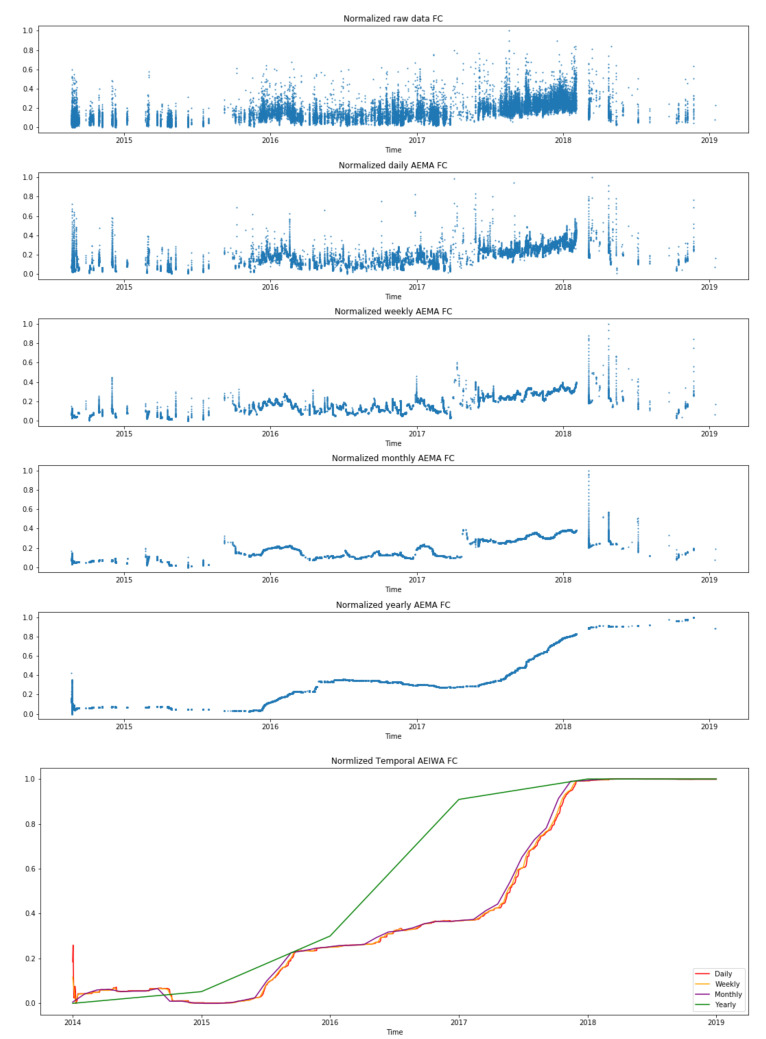
Absolute Error (AE) metrics of a single gas turbine using a FC autoencoder model with multiple time window representations of segmented moving average on the top and grouped incremental window on the bottom.

**Figure 5:**
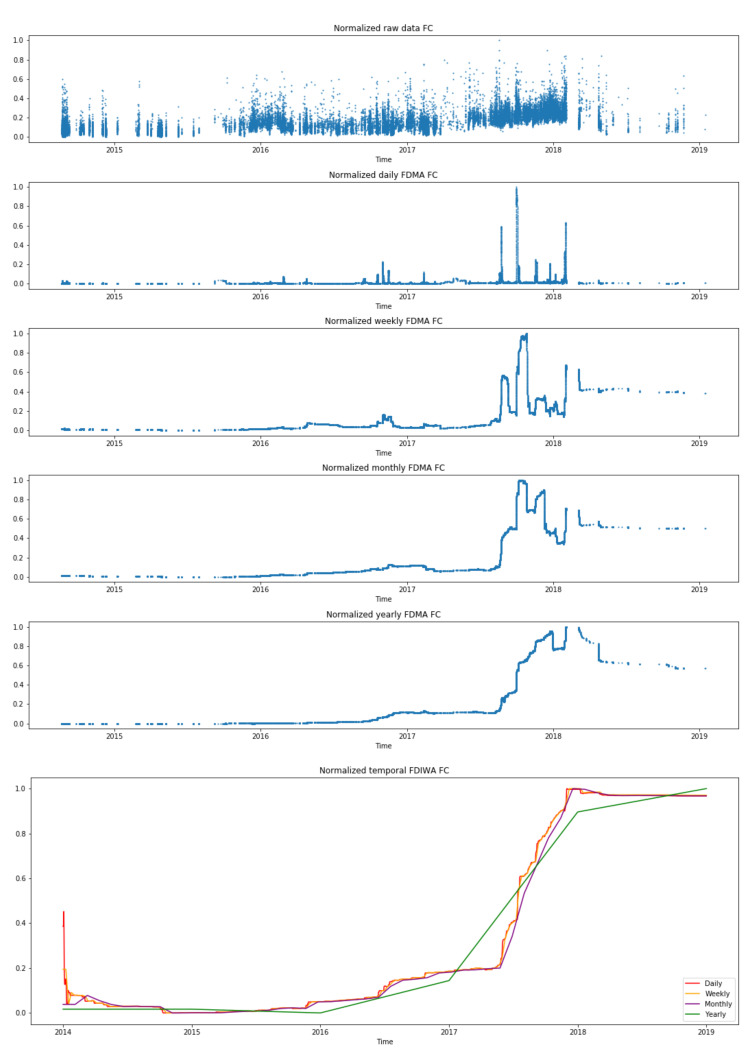
Fréchet Distance (FD) metrics of a single gas turbine using a FC autoencoder model with multiple time window representations of segmented moving average on the top and grouped incremental window on the bottom.

Figure 4 shows the values of AEMA for a single gas turbine’s compressor. The plot illustrates multiple time windows, where the larger the time-frequency, the smoother is its representation. Furthermore, it displays the same gas turbine component using the same distance (AE) but with the IWA calculation. In this case, the IWA calculation leads to a smoother function for all the time windows in comparison with the AEMA plots. Figure 5 shows the representation of the Fréchet distance (FD) using both averaging calculations, i.e., MA and IWA.

Both figures contain five subfigures using the time-window moving average, which are ordered from less to more granularity, plus a last subfigure with the IWA time-window. Therefore, in the top, the raw data are displayed (1 minute data); next, several time windows linked to the moving average are considered until the largest time window (yearly); finally, a last sixth subfigure plots several IWA results together.

From Figure 4, regarding the whole time-series of the different distances and calculations, a clear ascending trend (drift) is captured along time. Considering each time-series in detail, two main points are of interest due to its shift in the reconstruction error. First, at the end of 2015, and next, between 2017 and 2018. The yearly time window, as well as the AEIWA representations, shows clearly the incremental slope in these two periods. Nevertheless, these increments are also observable in the other representations as a slight shift. Therefore, for daily, weekly, and monthly time windows, the increments are from 0.0 to 0.2 and from 0.2 to 0.4, respectively. For the yearly time window, from 0.0 to 0.4 and from 0.4 to 0.8, respectively. Finally, the AEIWA representation shows the same shift location in all figures from 0.0 to 0.2 and from 0.4 to 1.0, respectively.

From Figure 5, most of the time-series present an ascending trend (drift), similar to those for the AE distance. However, the daily, weekly, and monthly time windows for the moving average calculation are only showing several significant shifts. Delving into these representations, the most relevant shifts are at the end of 2017 and at the beginning of 2018. In addition, a small shift is also displayed at the end of 2016. Ordered chronologically, these increases are from 0.0 to 0.2; from 0.2 to 0.5, then up to 1.0, its maximum; and the last shift is from 0.3 to 0.6. Observing the yearly time window for FDIWA, the increase is gradual, but higher slopes are observed at the same periods of time that for FDMA.

As shown in both figures, shifts have different slopes as well as importance; indeed, shift increments do not have the same dimension, but the drift is observed in all representations. The quantitative measurement of the gaps are highlighted to prove the deviation over time but their explanation are beyond the scope of this study.

## 4. Discussion

The objective of this study is to develop a soft sensor based on a data-driven machine learning technique following the autoencoder architecture for monitoring significant drift in the condition of the compressor in an industrial gas turbine. Changes have been studied through the combination of two different distances and time-window calculations analyzing the graphical evolution along time.

For the absolute error distance (see Figure 4), remarkable drift is observed in 2015/11 and 2017/05 in the moving average representation. Furthermore, in 2018/03, a significant drift jump can be observed. Finally, considering only the largest time window, the yearly time window, a general shift of the compressor performance is captured.

Next, in the incremental representation, similar patterns are also detected where events are revealed as a change of slope. Moreover, a whole assessment of the compressor behavior can also be done as in the yearly time window due to the nature of its calculation, the cumulative function, as shown in Equation (10). Indeed, the same ascending trend is also displayed. Therefore, this makes clear the drift in the behavior of the compressor in relation to the initial performance.

For the Fréchet distance (see Figure 5, the most significant changes regarding the moving average representation are shown in 2016/10, 2017/08, 2017/10, and 2018/03. According to the multiple time window, shifts are slightly moved aside due to the number of instances used to perform the distance computation for each case. The main drift jump is shown in 2017/10, but those in 2016/10 and 2018/03 are also significant enough. Some interesting changes are also shown in the yearly time window where a change in the general behavior is also displayed.

Then, the incremental representation does not follow the same pattern as in the absolute error distance, as only a significant change of slope is observed in 2017/08. Besides, a general drift of the behavior is displayed as well.

Only the 2018/03 event is seen using both distances. The main explanation is that the nature of the calculation is significantly different. The AE distance is computed considering the average of the error in a single time step, whereas the FD is the comparison of two multivariate distributions considering a fixed time window. These lead to different representations, as it is seen. The commonalities of the two distances is the ascending trend along time. Therefore, the drift in the behavior of the compressor in relation to the beginning of its performance becomes evident.

## 5. Conclusions

The goal of this study is to use an autoencoder architecture as a form of soft sensor to determine a drift between the short- and long-term performance of a system. As shown in the results, and further explained in the discussion, a qualitative indicator $\epsilon_{F}$ based on an autoencoder model approach is developed, showing a clear drift over gas turbine’s life.

The results have been underpinned by two different distance measurements as well as two different types of calculation, as detailed in Table 1. In all cases, the drift becomes evident. The causes of the drift may vary but the deterioration of the machine, the maintenance actions performed to the system, or the malfunctioning event of the equipment can be a rational explanation.

The focus of this research is put on the compressor because it is one of the main components of gas turbines, but this results would allow us to extrapolate the method to other components as well as the whole system.

For further research, a quantitative indicator can be obtained based on this soft sensor approach; detailed information about the maintenance, faults, and unexpected events can be combined with *Fresh Reconstruction discrepancy* to draw further results; and finally the same methodology will be used to assess the condition of other gas turbine’s components.

## Figures and Tables

**Table 1:** The combinations of the two considered distances and the two smoothing time window calculations.

	Moving Average	Incremental Window Average
Absolute Error	AEMA	FDMA
Fréchet distance	AEIWA	FDIWA

## Data Availability

Restrictions apply to the availability of these data. Data were obtained from Siemens Energy and are available from the authors with the permission of Siemens Energy.
